# Effect of high-flow nasal therapy on patient-centred outcomes in patients at high risk of postoperative pulmonary complications after cardiac surgery: a statistical analysis plan for NOTACS, a multicentre adaptive randomised controlled trial

**DOI:** 10.1186/s13063-022-06607-z

**Published:** 2022-08-20

**Authors:** Sarah N. Dawson, Yi-Da Chiu, Andrew A. Klein, Melissa Earwaker, Sofia S. Villar

**Affiliations:** 1 MRC Biostatistics Unit, School of Clinical Medicine, University of Cambridge, Cambridge, UK; 2 Papworth Trials Unit Collaboration, Royal Papworth Hospital NHS Foundation Trust, Cambridge, UK; 3 Department of Anaesthesia and Intensive Care, Royal Papworth Hospital, Cambridge, UK

**Keywords:** Statistical analysis plan, Adaptive design, Sample size re-estimation

## Abstract

**Background:**

The NOTACS trial will assess the efficacy, safety and cost-effectiveness of high-flow nasal therapy (HFNT) compared to standard oxygen therapy (SOT) on the outcomes of patients after cardiac surgery.

**Methods/design:**

NOTACS is an adaptive, international, multicentre, parallel-group, randomised controlled trial, with a pre-planned interim sample size re-estimation (SSR). A minimum of 850 patients will be randomised 1:1 to receive either HFNT or SOT. The primary outcome is days alive and at home in the first 90 days after the planned surgery (DAH90), with a number of secondary analyses and cost-effectiveness analyses also planned. The interim SSR will take place after a minimum of 300 patients have been followed up for 90 days and will allow for the sample size to increase up to a maximum of 1152 patients.

**Results:**

This manuscript provides detailed descriptions of the design of the NOTACS trial, and the analyses to be undertaken at the interim and final analyses. The main purpose of the interim analysis is to assess safety and to perform a sample size re-estimation. The main purpose of the final analysis is to examine the safety, efficacy and cost-effectiveness of HFNT compared to SOT on the outcomes of patients after cardiac surgery.

**Discussion:**

This manuscript outlines the key features of the NOTACS statistical analysis plan and was submitted to the journal before the interim analysis in order to preserve scientific integrity under an adaptive design framework. The NOTACS SAP closely follows published guidelines for the content of SAPs in clinical trials.

**Trial registration:**

ISRCTN14092678. Registered on 13 May 2020.

**Supplementary Information:**

The online version contains supplementary material available at 10.1186/s13063-022-06607-z.

## Background

High-flow nasal therapy (HFNT) has become increasingly used as a non-invasive form of respiratory support [1]. Patients typically tolerate it better than standard alternatives such as continuous positive airway pressure or non-invasive ventilation [2]. However, there is equipoise regarding its prophylactic use and effect on important patient-centred outcomes, hence the rationale for this trial. Recent systematic reviews in non-cardiac [3] and cardiothoracic [4] surgery concluded that HFNT could reduce respiratory support and pulmonary complications, and could be safely administered.

The NOTACS (Nasal High-Flow Oxygen Therapy After Cardiac Surgery) trial will assess the efficacy, safety and cost-effectiveness of HFNT on the outcomes of patients after cardiac surgery.

This paper outlines the statistical analysis plan (SAP) for the NOTACS trial, which has been submitted for publication prior to the interim analysis to uphold scientific integrity. Further details of the study can be found in the published NOTACS protocol [5]. The NOTACS SAP closely follows published guidelines for the content of SAPs in clinical trials [6].

### Objectives

The primary objectives are to determine if prophylactic use of HFNT (for a minimum of 16 h after tracheal extubation) is clinically and cost-effectively superior in comparison with standard oxygen therapy (SOT) up to 90 days after surgery, for adult patients undergoing cardiac procedures with cardiopulmonary bypass who are at high risk of postoperative pulmonary complications. The primary objective around clinical efficacy will be evaluated by comparing DAH90 (days alive and at home in the first 90 days after the planned surgery) between the two treatment arms.

The secondary objectives are to determine if prophylactic use of HFNT is able to:

- Reduce mortality, pulmonary complications, intensive care re-admission rate, length of hospital and intensive care stay.
- Reduce incidence of major complications including sepsis, acute kidney injury, myocardial infarction and stroke.
- Reduce readmission to hospital rate.
- Improve oxygenation as measured by the ROX Index.
- Improve patient-centred outcomes as measured using the EQ-5D-5L.
- Reduce patient level of assistance needed with activities of daily living as measured using BARTHEL questionnaire.
- Improve quality of survival as measured using ED-5D-5L quality-adjusted life years.
- Reduce health service and resource use.

## Methods/design

### Design and setting

The study is an adaptive, multicentre, parallel-group, randomised controlled trial with embedded cost-effectiveness analysis comparing the use of HFNT to SOT for a minimum of 16 h after tracheal extubation, in patients at high risk of respiratory complications following cardiac surgery. Eligibility criteria and further details of the study design and setting are provided in the published study protocol [5]. Patients will be recruited from the UK, Australia and New Zealand. The NOTACS study includes an interim sample size re-estimation (SSR) which is expected to take place in late 2022, when a minimum of 300 patients have been randomised and followed up for 90 days.

### Study protocol development and conduct

The NOTACTS trial was registered with ISRCTN (ISRCTN14092678) in May 2020. The trial is overseen by a Trial Steering Committee (TSC) and a Data Monitoring and Ethics Committee (DMEC).

### Randomisation and blinding

To reduce predictability, stratified random permuted blocks will be used to randomise patients, with block sizes of 4 or 6, stratified by centre. The allocation ratio of SOT to HFNT is 1:1.

Due to the nature of the intervention, clinical staff in ICU and on the wards cannot be blinded whilst the patient is receiving randomised therapy. However, a team of research staff at the central clinical trials unit will collect data on outcomes and these staff will be blinded. In addition, the decision to discharge patients from hospital, which affects the primary outcome, will be made by clinicians who are independent of the research team at each site, according to standard protocols. The interim analysis and SSR will be done by an independent unblinded statistician so that the trial statistician can remain blinded until the final analysis in order to preserve the type I error rate at 5%.

### Data storage and collection

Data will be kept on a bespoke data management database system, OpenClinica, with blinded and unblinded access. Anonymised patient data will be provided to the statistician by the data manager.

### Primary outcome

The primary outcome for the statistical analysis is DAH90.

### Secondary outcomes

The secondary outcomes include:

- DAH30 (days alive and at home in the first 30 days after the planned surgery)
- Incidence of adverse events and serious adverse events (including death)
- Incidence of stroke, sepsis, acute kidney injury and myocardial infarction
- Postoperative pulmonary complications
- ICU re-admission rate during index admission
- Total length of ICU stay (days) during index admission
- Total length of hospital stay (days) during index admission
- Re-admission to hospital
- Oxygenation, as measured by ROX Index
- Patient-reported outcomes (EQ-5D-5L)
- Patient level of assistance needed with Activities of Daily Living (BARTHEL questionnaire)
- Quality of survival (QALYs)
- Health service and resource use

### Sample size calculation

Results from the pilot study [7] and information provided by collaborative hospitals were used to derive the required sample size for the NOTACS study. The sample size calculation relied on several nuisance parameters (standard deviation of DAH90 in the SOT arm; standard deviation of DAH90 in the HFNT arm; treatment switch rate from SOT to HFNT; treatment switch rate from HFNT to SOT; overall drop-out rate; overall death rate) that were provided from the single centre pilot study or collaborative hospitals and may differ between sites in the multicentre design. The sample size calculation was performed by simulation due to the non-standard distribution of the primary end-point. Due to uncertainty in the nuisance parameters used and the sensitivity of the sample size calculation to changes in the nuisance parameters, the NOTACS study includes an interim SSR, a type of adaptive design. This will provide protection against important deviations from the initial assumptions in the original sample size calculation and ensure that the study is not underpowered.

DAH90 typically has a left-skewed bi-modal distribution with a small spike at 0 due to in-hospital deaths. The required sample size was obtained by simulations (100,000 replicates) by first generating length of stay (LOS) using a lognormal distribution. Based on the information provided by collaborating hospitals, the parameters of the lognormal distributions in both arms were derived through a pooled weighted average. The variability was calibrated to SD =12.85 in the control arm and SD=3.20 in the treatment arm. The median LOS in the control arm was set to 8 days. We assumed a 3% death rate (based on pilot data [7] and registry data [8]), and following the approach of Myles [9] we treated any death within the 90-day follow-up period as scoring 90 for LOS regardless of when the death occurred. LOS was truncated at 90 days (the maximum for our follow-up period). Finally, DAH90 was computed as 90 minus LOS. The resulting data are bimodal with a spike at 0, as seen with observed data of this type.

A total sample size of 310 has 90% power to detect an increase of 2 days in the median DAH90 using the Mann-Whitney-Wilcoxon test for the analysis. After adjustment for 12% crossover from standard oxygen to HFNT and 25% crossover from HFNT to standard oxygen as well as an extra 5% loss to follow-up (equally distributed among arms), the total sample size needed to detect a 2-day increase in DAH90 with 90% power with an intention-to-treat (ITT) analysis is 850 patients.

The minimum target sample size (based on initial assumptions) is 850 randomised participants. The final sample size target will be revisited after a minimum of 300 patients have been randomised and followed up to 90 days. The adaptive design will allow for a maximum sample size increase to 1152 patients. In the event that the interim analysis recommends a sample size less than 850, recruitment will continue to the original minimum target sample size of 850.

## Statistical analysis plan

This SAP describes the statistical analyses planned for the NOTACS trial. The health economic analyses are described separately in a health economics plan.

### Statistical principles

This SAP is based on version 3.0 (19 January 2022) of the trial protocol and version 0.43 (13th June 2022) of the trial SAP. The statistical analyses will be carried out using R (www.r-project.org). Other major statistical software may be used where appropriate. Derived variables are described in the additional document. Data will be checked for outliers and unexpected distributions or data points will be queried. Consistency checks between two or more variables will also be performed. Where relevant, variables will be summarised by the treatment arm using the following descriptive statistics: for continuous variables, the non-missing sample size, mean, standard deviation, median, maximum and minimum; for categorical variables, the frequency and percentages (based on the non-missing sample size) of observed levels will be reported.

### Handling of missing data

Withdrawal rates will be summarised by the treatment arm, and the timing of and reason for withdrawals will be reported. The proportion of missing data will be quantified by the treatment group for the variables included in the primary and secondary analyses. Based on the pilot study, the rate of missing data in DAH90 is expected to be low. Variables with >25% missing data may not be used in statistical regression modelling.

The trial was powered on the basis of a 5% loss to follow-up rate. Provided there is no more than 5% missing data in DAH90 and no obvious cause for concern over the pattern of missing data then we will run complete-case analysis. If the missingness rate in DAH90 exceeds 5%, we will take further steps to investigate the type of missingness for the subset of variables included in the primary and secondary analyses. If the missing data is found not to be MCAR in any instance, we will assume the missing data is MAR and perform multiple imputation as a sensitivity analysis to evaluate the robustness of the primary analysis. Our approach to assessing missing data and implementing multiple imputation is detailed in the additional document.

For derived variables such as DAH, the presence of missing data in the variables used to calculate these from may be more difficult to identify. DAH relies on participant location diaries to capture all changes in participant location with accurate start and end dates for each change in location. There may be instances where data is missing from participant location diaries despite it appearing that the participant location diary has been completed in full, if for instances a participant fails to record a change of location. This is a potential limitation of the DAH end-point. Where it is clear that a patient diary is incomplete, they will be completed where possible by calling the patient’s GP surgery and using hospital discharge summaries.

### Patient flow

The participant timeline can be found in the study protocol [5]. A CONSORT diagram will be produced as part of the statistical analysis to show the flow of patients through the study, from recruitment through to treatment allocation, discharge, 30 days and 90 days follow-up.

### Analysis populations

Each patient will be included or excluded from each of the analysis populations defined below. This will be carried out prior to unblinding to avoid bias.

#### Safety population

The safety population includes all subjects entered into the trial from the time of tracheal extubation up to 90 days after surgery (the period for which safety data is being collected) at the time of database lock. The safety population will be used to provide summary statistics on adverse events (AEs) and serious adverse events (SAEs), which will be reported by the treatment arm assigned during randomisation (or by the treatment arm actually received if it is different to that assigned during randomisation). The safety population will be examined at both the interim and final analyses. It will include patients with partial data as well as any patients who have withdrawn from the trial provided they continue to consent to their data being used.

#### Interim analysis population

The interim analysis population will include all patients who have been recruited at the time 300 patients have completed 90 days follow-up, including patients with partial data (e.g. anyone recruited after the 300th patient who has not yet completed their follow-up, and anyone recruited before the 300th patient who left the trial before completing follow up). If there is greater than 15% missing data for DAH90 (excluding missingness because of death, which is informative) and the interim analysis is delayed because of that, the interim analysis population may include more than 300 patients. The trial will recruit from the UK, Australia and New Zealand. It is expected that the majority of the patients recruited by the end of the trial will be from UK centres. To ensure that the results of the interim SSR reflect the expected proportions of UK, Australian and New Zealand recruits, we will seek to ensure that between 50 and 75% of the patients included in the interim analysis are recruited from the UK, with the remainder from Australia and New Zealand. This will ensure that even if differences exist between the countries, the effect will not disproportionately bias the interim SSR and therefore will not affect the power at the end of the trial. This requirement may impact on the timing of the interim analysis and may require that more than 300 patients have been randomised and followed up to 90 days to achieve the proportions stated.

#### Intention-to-treat population

The ITT population is the population that will be used for the majority of the analyses, including the analysis of the primary endpoint and the interim sample size re-estimation. The ITT population includes all subjects who were randomised, regardless of whether they received the treatment randomly allocated to them or completed follow-up. The data will be analysed assuming that the patient received the treatment they were randomly allocated to. If a patient dies after being randomised but before extubation occurs, they will be included in the ITT analysis population with a days at home score of zero.

#### Other populations

A per-protocol and two time-on-treatment (ToT) populations are also defined for use in sensitivity analyses. A full population is also defined. These are detailed in the additional document.

### Protocol deviations and adherence

There are two analysis populations relating to protocol non-adherence. The ITT analysis population includes all subjects who were randomised, regardless of whether they received the treatment randomly allocated to them or completed follow-up. The data will be analysed assuming that the patient received the treatment they were randomly allocated. The per-protocol population includes all subjects who adhered to the trial protocol by receiving the treatment randomly allocated for a minimum of 16 h no matter whether they complete all of their follow-ups. Any subjects who did not receive the treatment randomly allocated to them will be excluded from the per-protocol population.

Descriptive statistics of compliance variables will be reported at the final analysis, split by treatment arm. These will include:

- Summary of treatment compliance
- Reasons for non-compliance
- Summary of compliance for the ToT populations

### Baseline characteristics

Baseline data will be collected following consent. This will include demographic data (age, sex, residential status, etc.), past medical history, quality of life (EQ-5D-5L), activity of daily living (BARTHEL), health service and resource use questions, the EuroSCORE II and the ARISCAT score. Descriptive statistics summarised by the treatment arm will be reported.

### Interim analysis

The interim analysis will be performed after a minimum of 300 patients have been randomised and followed up for 90 days. If there is greater than 15% missing data for DAH90 (excluding missingness because of death, which is informative missingness) then the interim analysis may be delayed. If at the time 300 patients have been randomised and followed up, the rate of UK participation is outside the target range, the interim analysis may be delayed as per the definition of the interim analysis population.

Three main areas will be examined at the interim analysis:

- Safety
- Recruitment, compliance and data completeness
- SSR

The safety analyses at the interim analysis will be identical to the safety analyses at the final analysis, described below. Information on recruitment, treatment compliance, and data completeness will be reported at both the interim and final analysis. A summary of patient recruitment data will be presented by the centre, as well as by the treatment group and time or trial stage where appropriate. Compliance and data completeness will be summarised by the treatment group.

#### Sample size re-estimation

Due to uncertainty in the parameters used in the original sample size calculation and the sensitivity of the initial sample size calculation to changes in the nuisance parameters, NOTACS has been designed as an adaptive trial with an interim SSR planned after a minimum of 300 patients complete 90 days follow-up.

This sample size adaptation may prevent an underpowered trial if moderate deviations from the assumptions made for the initial sample size calculation are observed.

At the interim SSR, the accumulated data will be used to re-estimate a number of “nuisance” parameters including:

- SD of DAH90 in the SOT arm
- SD of DAH90 in the HFNT arm
- Treatment switch rate from SOT to HFNT
- Treatment switch rate from HFNT to SOT
- Overall drop-out rate
- Overall death rate

Treatment efficacy will not be assessed at the interim analysis.

An independent statistician will re-estimate the nuisance parameters using unblinded data (in order to allow the trial statisticians to remain blinded, and to preserve the type 1 error rate at 5%). The original sample size calculation will be repeated with the updated estimates to provide an updated sample size estimate which will be reported to the DMEC. The DMEC have the responsibility of agreeing the updated sample size following the rules in Table 1. Any recommendation outside of those listed in Table 1 should be clearly justified.

**Table 1:** Recommended sample size from interim SSR and course of action

Recommended sample size from interim SSR	Course of action
≤850	Continue recruitment to 850 patients
851–1152	Continue recruitment to the new recommended sample size
>1152	Continue recruitment to 1152 patients

No adjustments will be made to the significance level due to the interim analysis.

### Analysis for the primary endpoint

The analysis of the primary and secondary endpoints will take place at the end of the trial following database lock.

The primary outcome is DAH90. Following the approach of Myles et al., for the primary analysis, DAH90 will be treated as 0 for any patient that dies in the period between randomisation and the 90-day follow-up [9]. This definition is deemed appropriate on the basis that the death rate in the trial population is expected to be low (around 3%), most deaths are expected to occur within the initial hospital admission, the death rate is expected to be comparable between the two treatment arms, and it is not expected that the treatment will impact on the death rate. The primary analysis will be on the basis of intention to treat (ITT).

Due to the skewed nature of DAH scores, a Mann-Whitney-Wilcoxon test will be used for the primary efficacy analysis. Contrasts between treatment groups for DAH90 will be used to evaluate the difference in the median DAH90 at a 5% significant level. 95% confidence intervals giving a range of plausible effects will be reported. The non-parametric confidence interval will be calculated using either the Hodges-Lehmann method if an exact p-value is available, or a normal approximation otherwise.

The statistical analysis will be reported according to CONSORT extension guidelines for reporting of adaptive trials [10]. A review statistician will independently reproduce the final primary efficacy analysis.

### Analysis for secondary endpoints

Important clinical covariates and sub-groups will be included in exploratory secondary analyses, and will include:

- DAH90
- DAH30
- ARISCAT score
- EURROSCORE II
- Gender
- COPD
- Asthma
- Obesity (BMI>35kg/m^2^)
- Current smoking status
- Lower respiratory tract infection in last 4 weeks
- Age (≤ or > 80 years)
- First time or re-do surgery
- ROX index
- Extubation timing (≤ or >24 h after admission to ICU)
- Return to theatre (≤ or > 24 h of admission to ICU)
- Length of initial ICU stay
- Country
- Centre (UK sites only)

#### Secondary outcomes

A series of Mann-Whitney-Wilcoxon tests will be used to assess whether a treatment effect exists for: DAH30; length of ICU stay during index admission; ROX index at 2, 6, 12, 24 and 48 h post-extubation; individual countries; and individual centres (UK only). Difference in medians with corresponding 95% confidence intervals will be reported for each comparison. The non-parametric confidence interval will be calculated using either the Hodges-Lehmann method if an exact p-value is available, or normal approximation otherwise.

Chi-squared tests (or Fisher’s exact test where appropriate) will be used to assess the association between the treatment group with extubation timing (≤ or >24 h after admission to ICU) and return to theatre (≤ or > 24 h of admission to ICU).

To allow for the estimation of effect sizes and adjustment for potential clinically important covariates, quantile regression models will be fitted for DAH90 and DAH30 as outlined in Table 2. Quantile regression was chosen due to the heavily skewed nature of the DAH endpoints and due to the expectation of two spikes close to day 0 and day 90/30.

**Table 2:** Quantile regression models

Outcome variable	Explanatory variable(s)	Levels
DAH90	Treatment group	SOT [reference group], HFNT
DAH30	Treatment group	SOT [reference group], HFNT
DAH90	Treatment group ARISCAT risk category EUROSCORE II Gender COPD Asthma Obesity Current smoker Lower respiratory tract infection in last 4 weeks Age First time or re-do surgery Country	SOT [reference group], HFNT Low [reference group], intermediate, high - Female [reference group], Male No [reference group], Yes No [reference group], Yes No [reference group], Yes No [reference group], Yes No [reference group], Yes - First time [reference group], Re-do UK [reference group], Australia, New Zealand

### Sensitivity analyses

A number of sensitivity analyses will be performed to examine the robustness of the primary analysis. These include:

- Using the per-protocol population instead of the ITT population;
- Using each of the two ToT populations instead of the ITT population;
- Using an alternative definition of DAH90 for patients that die during follow-up;
- Examination of missing data, with multiple imputation of missing data where appropriate.

Further details of these sensitivity analyses can be found in the additional document.

### Safety analyses

All safety analyses will be performed on the safety population. Data on AEs and SAEs will be collected from the time of tracheal extubation to discharge. From discharge up to 90 days after surgery, only data on SAEs will be collected. Adverse events (AEs) and serious adverse events (SAEs) will be summarised separately. AE and SAE data will be listed by MedDRA Preferred Term and further grouped by System Organ Class. The frequencies of the AEs will be summarised by treatment groups. Death, stroke, sepsis, myocardial infarction and acute kidney injury are SAEs of special interest. Therefore, the rates of these will be estimated for each treatment group separately to the main SAE summary table.

### Other analyses

Descriptive statistics and summaries of recruitment and compliance will be examined at the final analysis (see additional document for details).

## Trial status and discussion

The NOTACS trial recruited its first participant in October 2020. Recruitment has been slower than expected due to the effects of the Covid-19 pandemic. Additional sites (including some international sites) have been added to the study in an attempt to mitigate the effects of the pandemic on patient recruitment. As of 20 July 2022, 267 patients have been randomised. The interim analysis is expected to take place in circa December 2022, when 300 patients have completed follow-up. This SAP was submitted to the journal before the interim analysis in order to preserve scientific integrity under an adaptive design framework.

## Supplementary Information

**Additional file 1.**

**Additional file 2.**

## Data Availability

The data that support the findings of this study will be made available following journal and funder requirements. Details of data accessibility will be included when the final results of the study are published.

## Abbreviations

AE: Adverse event
ARISCAT: A risk index for postoperative pulmonary complications
BARTHEL: A measure of performance in activities of daily living
CONSORT: Consolidated standards of reporting trials
COPD: Chronic obstructive pulmonary disease
DAH: Days alive and at home
DAH30: Days alive and at home in the first 30 days after the planned surgery
DAH90: Days alive and at home in the first 90 days after the planned surgery
DMEC: Data monitoring and ethics committee
EQ-5D-5L: A quality of life questionnaire
EUROSCORE: European system for cardiac operative risk evaluation
GP: General practitioner
HFNT: High-flow nasal therapy
ICU: Intensive care unit
ISRCTN: International standard randomised controlled trial number
ITT: Intention-to-treat
LOS: Length of stay
MAR: Missing at random
MCAR: Missing completely at random
MedDRA: Medical dictionary for regulatory activities
NOTACS: Nasal High-Flow Oxygen Therapy After Cardiac Surgery
QUALY: Quality-adjusted life years
ROX: Respiratory rate oxygenation
SAE: Serious adverse event
SAP: Statistical analysis plan
SD: Standard deviation
SOT: Standard oxygen therapy
SSR: Sample size re-estimation
TSC: Trial steering committee
ToT: Time-on-treatment
UK: United Kingdom
