# Supplementary material for: Effect of high-flow nasal therapy on patient-centred outcomes in patients at high risk of postoperative pulmonary complications after cardiac surgery: a statistical analysis plan for NOTACS, a multicentre adaptive randomised controlled trial
Source: Trials. 2022 Aug 20;23:699. doi: 10.1186/s13063-022-06607-z (PMC9391633; doi:10.1186/s13063-022-06607-z)
Supplement: Supplementary file 1 — Additional file 1. [file 13063_2022_6607_MOESM1_ESM.docx]

**Additional document**

**Derived variables**

**Days at home**

For patients alive at time t, DAH is derived as follows:

$$DAH_{t}=t- \sum_{j=1}^{J} {LOS}_{j}-I_{n}\sum_{i=1}^{I} S_{i}$$

$t$ represents a follow-up time point. $J$ represents the total number of hospital admissions until time $t$, ${LOS}_{j}$ is the total length of stay associated with admission $j$ (except for j=1, where LOS is calculated from the date of surgery and not the date of admission), $I_{n}$ is a binary variable that takes the value 1 if the original residence of that patient was not a nursing centre, $I$ represents the total number of nursing centre admissions until time $t$, $S_{i}$ is the total length of stay associated with admission $i$ to a nursing centre. Patients that are not alive at time t will be imputed with a DAH of 0 (1).

Where a participant changes location (e.g. hospital to home, or home to hospital) their location for that day is based on where they spent the night. Specifically, if a patient changes location on day 30 or day 90 it will be incorporated in the DAH score based on where the patient spent the night.

Where a patient chooses to stay at the home of a relative or friend or in paid accommodation away from home (e.g. a hotel) for social reasons only and there is no additional care or support provided, this will be considered as “home” for the purpose of calculating DAH.

Where a patient stays at the home of a relative or friend or in paid accommodation away from home (e.g. a hotel) and additional care or support is provided, this will be considered as time spent away from home for the purpose of calculating DAH.

### Time spent on intervention and Compliance

Time spent on SOT or HFNT will be calculated for each patient using the oxygen log case report form (CRF). The date and time started and the date and time stopped are captured within the oxygen log CRF. Changes to oxygen therapy settings are also recorded with start and stop dates and times.

In straightforward cases, the time spent on either SOT or HFNT can be derived simply by the modulus of the time difference in minutes between the date and time treatment was started and stopped.

For patients with multiple start and stop dates and times, or for patients where the intervention settings were changed, the time spent on intervention can be derived by taking the modulus of the time difference in minutes between the date and time treatment was started and stopped for each individual entry on the oxygen log CRF, and summing across these for each patient to get a total time in minutes.

When patients are transferred from one area of the hospital to another they may be placed onto portable oxygen temporarily. For the purpose of determining treatment compliance, patients will be considered as compliant provided that they receive 16 hours of randomised therapy and that no period off of randomised therapy (e.g. when on portable oxygen therapy whilst a patient is moved within a hospital) exceeds 1 hour.

To allow for patients who change from SOT to HFNT or vice versa, time on SOT and time on HFNT will both be calculated separately for each patient.

### Age

Age will be defined as age at baseline. Age will be calculated in years, as the difference between the date the baseline CRF was completed and the date of birth. Age will be rounded down to whole years (e.g. 50.65 years will be rounded down to 50 years).

### ROX Index

Data to calculate ROX Index will be collected at 2, 6, 12, 24 and 48 hours post extubation. It is calculated by the ratio of SpO_2_/FiO_2_ (%) divided by respiratory rate (breaths per minute).

### EuroSCORE

Care data will be collected to calculate the EuroSCORE II. This will be recorded in the database as a single, pre-calculated percentage with no further derivation needed.

### ARISCAT

Data will be collected to calculate the ARISCAT score. ARISCAT score will be recorded in the database as 7 individual components, from which we will calculate the ARISCAT score using the following scoring system (2) (3):

| **ARISCAT component** | **Level** | **Score** |
| --- | --- | --- |
| Age | ≤50 | 0 |
|  | 51-80 | +3 |
|  | >80 | +16 |
| Pre-operative SpO2 | ≥96% | 0 |
|  | 91-95% | +8 |
|  | ≤90% | +24 |
| Respiratory infection in the last month | No | 0 |
|  | Yes | +17 |
| Preoperative anemia (Hgb ≤10 g/dL) | No | 0 |
|  | Yes | +11 |
| Surgical incision | Peripheral | 0 |
|  | Upper abdominal | +15 |
|  | Intrathoracic | +24 |
| Duration of surgery | <2 hours | 0 |
|  | 2-3 hours | +16 |
|  | >3 hours | +23 |
| Emergency procedure | No | 0 |
|  | Yes | +8 |

A patient receives one score for each of the seven ARISCAT components. The overall ARISCAT score is calculated by summing the scores for the seven ARISCAT components for each patient.

ARISCAT score can also be categorised into three risk categories such that:

- Low risk: ARISCAT score <26
- Intermediate risk: ARISCAT score 26-44
- High risk: ARISCAT score >44

### EQ-5D-5L and QALYs

The mapping utility value of EQ-5D-5L will be created through crosswalk index value sets (4) or new EQ-5D-5L value sets should these become available, and QALYs will be calculated at 30 days and 90 days after surgery. This will be done by the health economist.

### BMI

Body mass index (BMI) will be calculated using the following formula:

$$BMI=\frac{m}{h^{2}}$$

Where *m* = mass (in kilograms)

*h* = height (in meters)

### Procedure length

Procedure length will be calculated as the time of procedure end minus the time of procedure start, and will be calculated in minutes.

### Time with cross-clamp on

Time with cross-clamp on will be calculated as the time of cross-clamp coming off minus the time of cross-clamp going on, and will be calculated in minutes.

### Time in inpatient locations

For the index admission, the time spent in a number of inpatient locations (ward, critical care unit, recovery unit, etc) will be calculated as the difference in minutes between the date and time a patient left a location minus the date and time a patient entered a location.

For example, if a patient entered the critical care unit at 3:15pm on 1^st^ January 2021 and left the critical care unit at 7:20am on 2^nd^ January 2021, their time spent on the critical unit would be 965 minutes (or 16 hours and 5 minutes). If the same patient then arrived on a ward at 7.30am on 2^nd^ January 2021 and left the ward at 6:45pm on 4^th^ January 2021, their time spent on the ward would be 3555 minutes (or 2 days 11 hours and 15 minutes).

**Populations**

In addition to the populations defined in the manuscript, the following populations will be included in the statistical analysis:

**Full population**

The full population includes all patients recruited to the trial. In the case of constructing a CONSORT diagram, in this instance only, the full population will also include those screened for the trial.

**Per-protocol population**

The per-protocol population includes all subjects who adhered to the trial protocol by receiving the treatment randomly allocated to them for a minimum of 16 hours no matter whether they completed all of their follow-ups. Any subject who did not receive the treatment randomly allocated to them for at least 16 hours will be excluded from the per-protocol population.

For the purpose of determining treatment compliance, patients will be considered as compliant provided that they receive 16 hours of randomised therapy and that no period off of randomised therapy exceeds 1 hour.

**Time-on-treatment populations**

Two time-on-treatment (ToT) populations will be defined in a similar way to the per-protocol population, but with alternative minimum treatment periods. These will include all subjects who received the treatment they were randomly allocated to for:

1. At least 8 hours
2. At least 24 hours

For the purpose of determining the ToT populations, patients will be considered as compliant provided that they receive either 8 hours (ToT population A) or 24 hours (ToT population B) of randomised therapy and that no period off of randomised therapy exceeds 1 hour.

The analysis of ToT population B will only be performed if the sample size for this population is at least 8 (regardless of treatment group assignment). This is because it is not possible to obtain a statistically significant result with 7 or fewer samples in a Mann-Whitney-Wilcoxon test.

**Sensitivity analysis**

**Alternative population definitions**

The primary efficacy analysis will be repeated with a number of alternative population definitions to examine the robustness of the results from the primary efficacy analysis. These alternative analyses will include:

- Using the per-protocol population instead of the intention-to-treat population
- Using each of the two time-on-treatment populations instead of the intention-to-treat population

**Alternative definition of DAH90 for patients that die during follow-up**

In the primary efficacy analysis DAH90 will be treated as 0 for any patient that dies during the trial, following the methods of Myles (1). We will perform a sensitivity analysis for the primary efficacy analysis to assess the impact of assigning a DAH90 score of zero to patients that die at any time within the 90 day follow-up period by relaxing this rule and replacing these zero values by the observed DAH90 value for these patients. In this sensitivity analysis, DAH90 will be calculated as post-randomisation-to-death duration – LOS, where post-randomisation-to-death duration ≤90.

**Missing data**

If the missingness rate in DAH90 exceeds 5%, we will take further steps to investigate the type of missingness for the subset of variables included in the primary and secondary analyses. We will use logistic regression to investigate whether the missingness indicator of each of these variables can be predicted by the other variables. If none of the missing indicators can be predicted by the other variables (i.e. p>0.05) we will assume that these data are missing completely at random (MCAR). Otherwise we will assume that the missing data are missing at random (MAR). We will also use Little’s test (5) for the continuous variables as a further way to assess the MCAR assumption. If the observed missing data are found to be MCAR in all of the missing data tests, we will ignore missing observations and run complete-case analysis.

If the missing data mechanism is found not to be MCAR in any of the missing data mechanism tests, as a sensitivity analysis to evaluate the robustness of the primary analysis we will assume the missing data mechanism is MAR and impute the missing data by using multiple imputation by chained equations (6). The number of imputed data sets will be based on the percentage of patients with missing data in the subset of variables included in the missing data mechanism tests (7). For example, if 5% of patients have missing data in the subset of variables included in the missing data mechanism tests then five imputed data sets will be produced. For each of these *m* imputed datasets, ten imputation cycles will be used (7). The imputed values from the last cycle in each imputed data set will be retained and these will form our *m* imputed data sets. The imputation models will include the primary outcome variable and relevant explanatory variables used in the primary analysis and secondary analyses sections of the final analysis report to prevent estimation bias (7). In the imputation model we will use predictive mean matching for all covariables except for derived variables. For derived variables we will use a fixed formula to impute missing data. The primary analysis will be repeated for each of the *m* imputed data sets, and the median p-value across these *m* individual analyses will be calculated (8). The median p-value will be provided as the result of this sensitivity analysis.

As the main focus of the missing data analysis is on the robustness of the primary analysis, if the proportion of missing data for secondary analysis variables is more than 5% but the proportion of missing data for the primary endpoint is less than 5%, no further investigations into the missing data mechanism or imputation will be performed.

The way missing data is handled may be different for health economic purposes, as imputation may be needed even if the proportion of missing data is low to avoid exclusion of patients that partially contribute data. This will be detailed separately in the health economics analysis plan (HEAP).

**Other analyses**

**Descriptive statistics**

Descriptive statistics will be reported at the final analysis for a range of variables, split by treatment arm. These will include:

- Missing data for primary and secondary analysis variables
- Baseline demographic variables
- Baseline ethnicity, separately for UK, Australia and New Zealand
- Chosen follow-up method
- Patient-related risk factor for postoperative pulmonary complications
- Baseline residence and support requirements
- Past medical history
- Baseline surgical intervention plan
- Baseline EUROSCORE II and ASA risk rating
- Baseline ARISCAT score
- Pre-operative blood results
- Pre-admission medications
- Surgical procedure
- Oxygen log
- Post-operative blood gas on intervention
- Post-operative blood gas post intervention
- Post-operative blood results post extubation
- Post-extubation ROX index
- Return to theatre
- Diagnostic tests completed after tracheal extubation to discharge
- 30 day resource use
- 90 day resource use
- Discharge Participant and Family Resource Use questionnaire
- DAH30
- DAH90
- Duration (days) of hospital stay for index admission following surgery
- Time spent at each inpatient location for index admission
- Inpatient medication
- Medication after discharge from index admission
- Discharge destination
- Health professional visits after tracheal extubation
- Hospital re-admission following discharge
- Duration (days) of all hospital stays (including both index admission and any re-admissions)
- Frequency and duration (days) of non-hospital stays away from home following discharge from index hospital admission
- Baseline EQ-5D-5L questionnaire
- Discharge EQ-5D-5L questionnaire
- Baseline Barthel Index questionnaire
- Discharge Barthel Index questionnaire

**Recruitment**

A CONSORT diagram will show the flow of patients through the trial, from recruitment through to treatment allocation and the three follow-up time-points (discharge, 30 days and 90 days).

Descriptive statistics of recruitment variables will be reported at the final analysis, split by treatment arm. These will include:

- Inclusion and exclusion criteria
- Eligibility
- Number of patients at each study site by study stage
- Number of patients completing 90 day follow-up by country
- Withdrawals

**Compliance**

Descriptive statistics of compliance variables will be reported at the final analysis, split by treatment arm where appropriate. These will include:

- Summary of treatment compliance
- Reasons for non-compliance
- Summary of compliance for the time-on-treatment populations

# References

1. *Validation of days at home as an outcome measure after surgery: a prospective cohort study in Australia.* Paul S Myles, Mark A Shulman, Stephane Heritier, Sophie Wallace, David R McIlroy, Stuart McCluskey, Isabella Sillar, Andrew Forbes. s.l. : BMJ Open, 2017, Vol. 7.

2. *Prediction of Postoperative Pulmonary Complications in a Population-Based Surgical Cohort.* Jaume Canet, Lluís Gallart, Carmen Gomar, Guillem Paluzie, Jordi Valle, Jordi Castillo, Sergi Sabate, Valentín Mazo, Zahara Briones, Joaquín Sanchis. s.l. : Anesthesiology, 2010, Vol. 113, pp. 1338 –1350.

3. *Prospective external validation of a predictive score for postoperative pulmonary complications.* Mazo V, Sabaté S, Canet J, Gallart L, de Abreu MG, Belda J, Langeron O, Hoeft A, Pelosi P. 2, s.l. : Anesthesiology, 2014, Vol. 121, pp. 219-231.

4. *Interim Scoring for the EQ-5D-5L: Mapping the EQ-5D-5L to EQ-5D-3L values sets.* al, Van Hout B et. s.l. : Value in Health, 2012, Vol. 15, pp. 708-715.

5. *A Test of Missing Completely at Random for Multivariate Data with Missing Values.* Little, R.J.A. s.l. : Journal of the American Statistical Association, 1988, Vol. 83, pp. 1198-1202.

6. *Multiple imputation by chained equations: what is it and how does it work?* Azur MJ, Stuart EA, Frangakis C, Leaf PJ. 1, s.l. : Int J Methods Psychiatr Res, 2011, Vol. 20, pp. 40-49.

7. *Multiple imputation using chained equations: Issues and guidance for practice.* Ian R. White, Patrick Royston, Angela M. Wood. 4, s.l. : Statist. Med, 2010, Vol. 30, pp. 377-399.

8. *Methods for significance testing of categorical covariates in logistic regression models after multiple imputation: power and applicability analysis.* Eekhout, I., van de Wiel, M.A. & Heymans, M.W. s.l. : BMC Med Res Methodol, 2017, Vol. 17, p. 129.
