# Supplementary material for: Effect of high-flow nasal therapy on patient-centred outcomes in patients at high risk of postoperative pulmonary complications after cardiac surgery: a statistical analysis plan for NOTACS, a multicentre adaptive randomised controlled trial
Source: Trials. 2022 Aug 20;23:699. doi: 10.1186/s13063-022-06607-z (PMC9391633; doi:10.1186/s13063-022-06607-z)
Supplement: Supplementary file 2 — Additional file 2. [file 13063_2022_6607_MOESM2_ESM.docx]

**Appendix 1**

The NOTACS study team members (in alphabetical order) (please tag in Pubmed):

Duckworth M, Papworth Trials Unit Collaboration, Royal Papworth Hospital, Cambridge, UK

Fox-Rushby J, King’s College London, London, UK

Kunst G, King’s College London, London, UK

Litton E, Intensive Care Unit, Fiona Stanley Hospital, Perth, Western Australia, Australia

Martinez G, Papworth Trials Unit, Royal Papworth Hospital, Cambridge, UK

Murphy G, University of Leicester, Leicester, UK

Parke R, Auckland District Health Board, Auckland, New Zealand

Shetty S, King’s College London, London, UK

Zochios V, University Hospitals Birmingham, Birmingham, UK
